# Diabetes Mellitus and Obesity as Risk Factors for Bladder Cancer Prognosis: A Systematic Review and Meta-Analysis

**DOI:** 10.3389/fendo.2021.699732

**Published:** 2021-10-07

**Authors:** Yu Lu, Jing Tao

**Affiliations:** Department of Interventional Radiotherapy, Huzhou Central Hospital, Affiliated Central Hospital Huzhou University, Huzhou, China

**Keywords:** urinary bladder cancer, diabetes, overweight, obesity, all-cause mortality, cancer specific mortality, disease progression, disease recurrence

## Abstract

**Background:**

Urinary bladder carcinoma is common in developed settings, and prognosis may be impacted by lifestyle factors such as excess body weight and diabetes mellitus. The present meta-analysis aimed to systematically collate and analyze evidence on the impact of diabetes and excess BMI on bladder cancer outcomes.

**Methods:**

PubMed, Scopus, and Google Scholar databases were screened for relevant studies that examined the association between bladder cancer outcomes and diabetes and/or excess body weight. The primary outcomes for this study were mortality (both all-cause and cancer-specific), risk of cancer progression, and recurrence. Strength of association was presented in the form of pooled adjusted hazard ratios (HR). Statistical analysis was performed using STATA version 16.0.

**Results:**

Twenty-five articles met inclusion criteria. Nine of these examined diabetes mellitus while 16 studied body mass index. All studies were retrospective. Diabetic patients had significantly higher risk for all-cause mortality (HR 1.24, 95% CI: 1.07, 1.44, n=3), cancer specific mortality (HR 1.67, 95% CI: 1.29, 2.16, n=7), disease progression (HR 1.54, 95% CI: 1.15, 2.06, n=8), and recurrence (HR 1.40, 95% CI: 1.32, 1.48, n=8) compared to non-diabetics. No statistically significant risk change for all-cause mortality, cancer specific mortality, disease progression, and recurrence was found for overweight patients. However, obese individuals were at higher risk for disease progression (HR 1.88, 95% CI: 1.41, 2.50, n=3) and recurrence (HR 1.60, 95% CI: 1.06, 2.40, n=7) compared to normal BMI patients.

**Conclusions:**

These findings suggest that diabetes and excess body weight negatively influences bladder cancer prognosis and outcome. The increased risk of mortality due to diabetes was similar to that in the general population. Since retrospective studies are potentially susceptible to bias, future prospective studies on this subject are required.

## Introduction

Urinary bladder cancer is quite prevalent, particularly in high-income settings (1). Bladder cancer is categorized as muscle invasive and non-muscle invasive: muscle invasive bladder cancers have low 5-year survival rates of ~35-40%, while non-muscle invasive bladder cancers have much higher survival rates (89-98%) accompanied by high five-year progression (~5-20%) and recurrence rates (~28-50%) (2–5). The resection of recurring tumors and subsequent treatment is often required, and expenditures associated with bladder cancer are often elevated compared to other cancers (6, 7). As such, frequent follow-up is advised for all bladder cancer patients post-treatment.

Efforts have been made to predict prognosis in bladder cancer patients using scoring tables (8). These tables focus on primary tumor characteristics. However, lifestyle factors such as obesity, smoking, as well as the presence of diabetes mellitus, can affect prognosis and modify follow-up schedules. Both diabetes mellitus and obesity are increasing in prevalence globally (9, 10), and insulin resistance and hyperinsulinemia have been proposed to affect bladder cancer risk and prognosis (11–13). Although several studies have looked at the link between body mass index and diabetes with cancer risk, few have looked at the impact of these factors on overall survival, tumor recurrence, and progression. Therefore, this current study aimed to pool available information and assess the impact of diabetes and elevated BMI on bladder cancer outcomes through a meta-analysis.

## Materials and Methods

### Search Strategy

The literature search was designed and conducted based on PRISMA (Preferred Reporting Items for Systematic Reviews and Meta-analyses) guidelines. We screened PubMed, Scopus, and Google Scholar academic databases for all English-language publications published prior to March 31, 2021. The search strategy incorporated medical topic heading (MeSH) terminology and free text words (**Supplementary Table 1**). The search aimed to identify studies reporting on the association of bladder cancer mortality, progression, and recurrence with diabetes and/or high body mass index. The primary outcomes for this meta-analysis were mortality (both all-cause and cancer specific), risk of cancer progression, and recurrence.

### Selection Criteria and Methods

Study titles and abstracts were initially reviewed by two subject experts. Following this, the full texts for candidate studies were subsequently reviewed. Disagreements were resolved through discussion. Only studies that met all inclusion criteria were included for meta-analysis. Reference lists from included studies were manually screened for additional candidate studies.

**Inclusion Criteria:** To be included, studies must have been either retrospective record-based studies or prospective and have examined the impact of diabetes and/or high body mass index on bladder cancer outcomes.

**Exclusion Criteria:** Case reports, review articles, and other such studies were excluded. Studies that did not provide data on the outcomes of interest or did not examine the exposures of interest (diabetes or body mass index) were excluded.

### Data Extraction and Quality Assessment

Relevant data from included studies was extracted using a set form by two independent reviewers. Extracted data included identification details, study setting, study design, sample size, follow-up duration, and main findings. Study quality was assessed using the Newcastle-Ottawa Quality Assessment Scale (14).

### Statistical Analysis

This meta-analysis was conducted using STATA software (version 16.0) and reported effect sizes as pooled hazard ratios with 95% confidence intervals (CIs). Separate analyses were performed for diabetes and body mass index. Subgroup analysis was performed for tumor type (muscle invasive or non-muscle invasive), tumor stage, and tumor grade. I^2^ was used to denote heterogeneity. We used random effects model as the included studies were diverse in their characteristics i.e., study subjects, geography, ethnicity, tumor characteristics etc. (15). P values under 0.05 was taken as statistically significant. Egger’s test was employed to assess for publication bias.

## Results

### Selection of Articles, Study Characteristics and Quality of Included Studies

**Figure 1:**
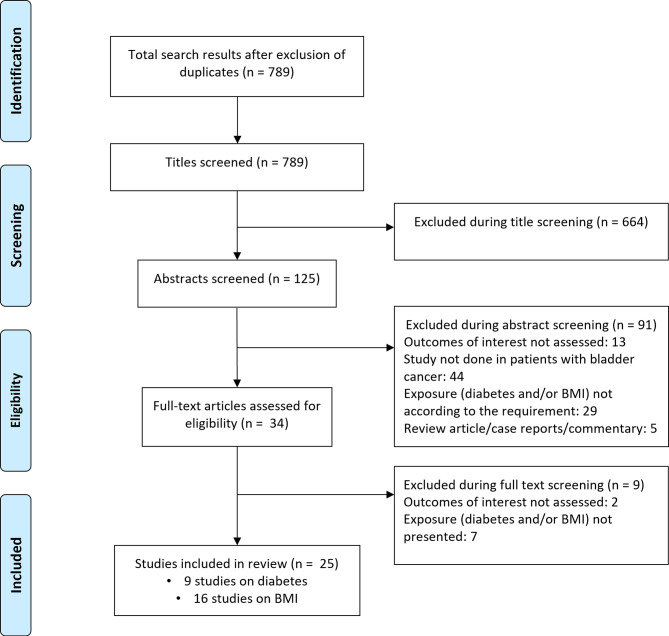
Study inclusion process.

Literature search revealed 789 candidate studies (**Figure 1**). Title screening removed 664 studies, and 91 were removed after abstract screening. The remaining 34 studies were then reviewed in detail, ultimately leaving 25 studies for inclusion in the meta-analysis. Nine of these studies focused on diabetes mellitus while 16 investigated body mass index (16–40) (**Supplementary Tables 2**, **3**). Of the nine studies that documented the link between diabetes and bladder cancer outcomes, three were conducted in South Korea, two in Taiwan, and one each in the Netherlands and USA. The remaining two studies were multicentered. Eight of the nine studies were retrospective analyses of patient data. Of the sixteen studies that investigated the link between body mass index and bladder cancer patient outcomes, three were performed in the USA, two in China, and one each in Germany, France, Turkey, Canada, and South Korea. The remaining six studies were multicentered. All sixteen studies were retrospective analyses of patient data. Study quality was good overall (**Supplementary Table 4**), with the majority of studies reporting appropriate processes for participant selection, outcome ascertainment, and controls.

### Association Between Diabetes and Bladder Cancer Outcome

**Figure 2:**
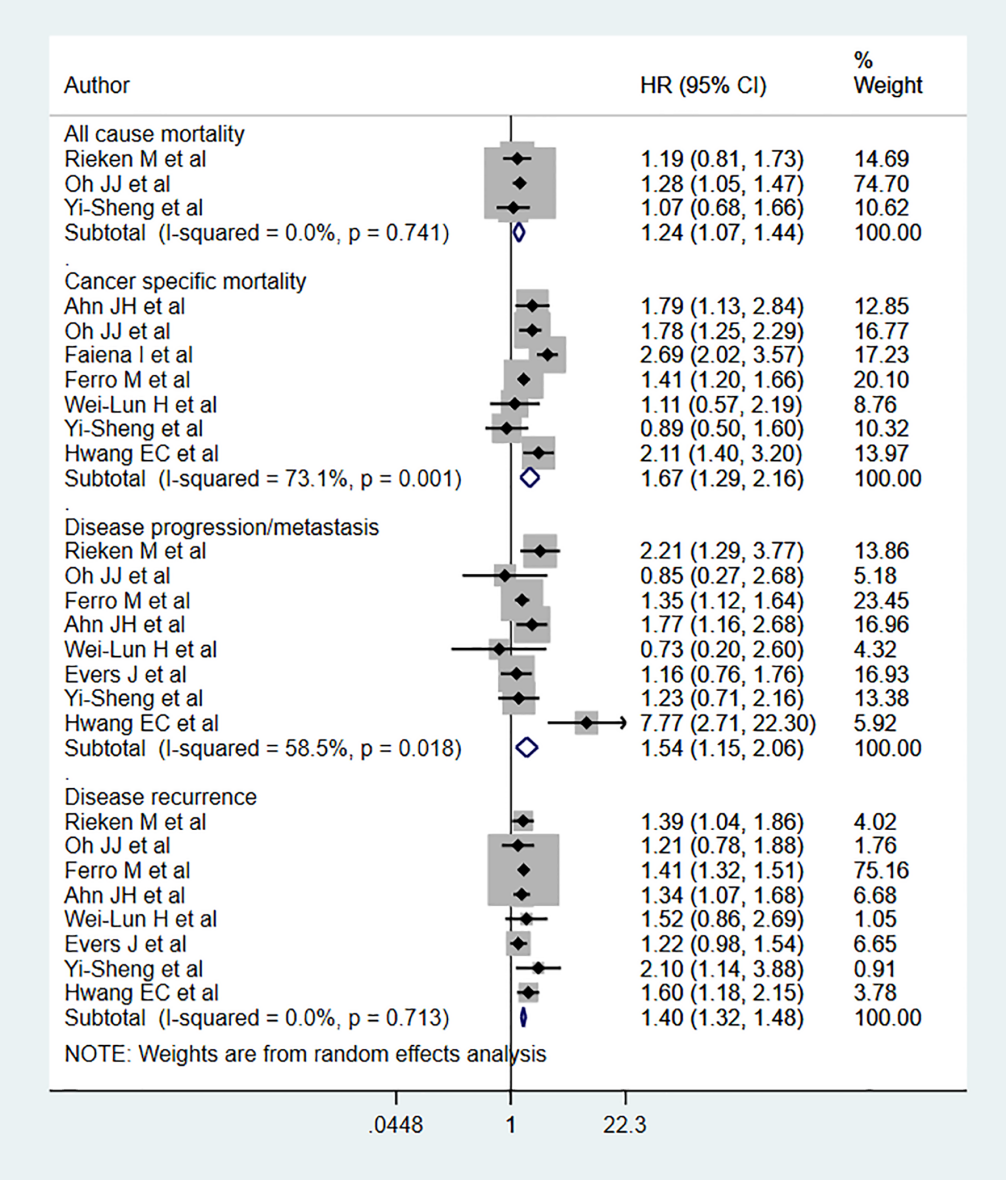
Relationship between diabetes and bladder cancer outcomes.

Diabetic patients had significantly higher risks for both all-cause mortality (HR 1.24, 95% CI: 1.07, 1.44, n=3) and cancer-specific mortality (HR 1.67, 95% CI: 1.29, 2.16, n=7) than non-diabetics (**Figure 2**). Diabetic patients also had higher risks of disease progression (HR 1.54, 95% CI: 1.15, 2.06, n=8) and recurrence (HR 1.40, 95% CI: 1.32, 1.48, n=8) compared to non-diabetics (**Figure 2**). For disease progression as an outcome, we ran the analysis after excluding the study by Hwang EC et al. (24) and found the association to be still significant but comparatively lower in magnitude (HR 1.40, 95% CI: 1.17, 1.67, n=7). Egger’s test did not show any evidence of publication bias for any of the examined outcomes (P=0.65 for all-cause mortality; P=0.43 for cancer specific mortality, P=0.72 for progression, and P=0.17 for recurrence).

Subgroup analysis showed that all-cause mortality risk among diabetics with an advanced tumor stage (≥T2) (HR 1.25, 95% CI: 1.07, 1.47, n=2) was elevated (**Table 1**). Cancer-specific mortality risk was high among diabetics with both early (PTa or T1) (HR 1.43, 95% CI: 1.23, 1.66, N=3] and advanced-stage tumors (HR 1.58, 95% CI: 1.04, 2.39, n=3). Disease progression risk was high in diabetics with early-stage tumors (HR 1.45, 95% CI: 1.16, 1.82, n=5), while recurrence risk was elevated in diabetics with both early (HR 1.39, 95% CI: 1.31, 1.48, N=5) and advanced-stage tumors (HR 1.54, 95% CI: 1.20, 1.97, n=3) (**Table 1**).

**Table 1:** Subgroup analysis for diabetes as a bladder cancer risk factor.

	Pooled effect size (Hazard ratio; HR) (95% Confidence Interval)			
	Stage of tumor		Grade of tumor	
	Early stage (PTa or T1)	Advanced (≥T2)	Low	High
All-cause mortality	N=1	N=2	—	N=3
	1.19 (0.81, 1.74)	1.25 (1.07, 1.47)		1.24 (1.07, 1.44)
Cancer specific mortality	N=3	N=3	N=2	N=4
	1.43 (1.23, 1.66)	1.58 (1.04, 2.39)	1.96 (1.44, 2.67)	1.39 (1.11, 1.76)
Risk of progression	N=5	N=3	N=3	N=5
	1.45 (1.16, 1.82)	1.97 (0.59, 6.58)	2.13 (1.00, 4.53)	1.39 (1.09, 1.76)
Risk of recurrence	N=5	N=3	N=3	N=5
	1.39 (1.31, 1.48)	1.54 (1.20, 1.97)	1.34 (1.17, 1.55)	1.41 (1.32, 1.51)

Out of the 9 studies included in the meta-analysis, only one study had subjects with muscle invasive bladder. Similarly, in only one study, majority of the subjects had tumor size>3 cm; In all the studies, patients did not have >2 tumors and none of the studies reported presence of carcinoma in situ in majority of the subjects. The modality of treatment in almost all the studies was transurethral resection of bladder with/without adjuvant therapy. Therefore, due to lack of variation for these variables among the included studies, sub-group analysis was not conducted on these variables.

The risks of cancer-specific mortality (Low grade: HR 1.96, 95% CI: 1.44, 2.67, n=2; High grade: HR 1.39, 95% CI: 1.11, 1.76, n=4), disease progression (Low grade: HR 2.13, 95% CI: 1.00, 4.53, N=3; High grade: HR 1.39, 95% CI: 1.09, 1.76, N=5], and disease recurrence (Low grade: HR 1.34, 95% CI: 1.17, 1.55, n=3; High grade: HR 1.41, 95% CI: 1.32, 1.51, n=5) were elevated among diabetics with either low or high grade tumors. For all-cause mortality, risk was elevated only among diabetics with high grade tumors (HR 1.24, 95% CI: 1.07, 1.44, n=3) (**Table 1**).

### Association Between Body Mass Index (BMI) and Bladder Cancer Outcome

**Figure 3:**
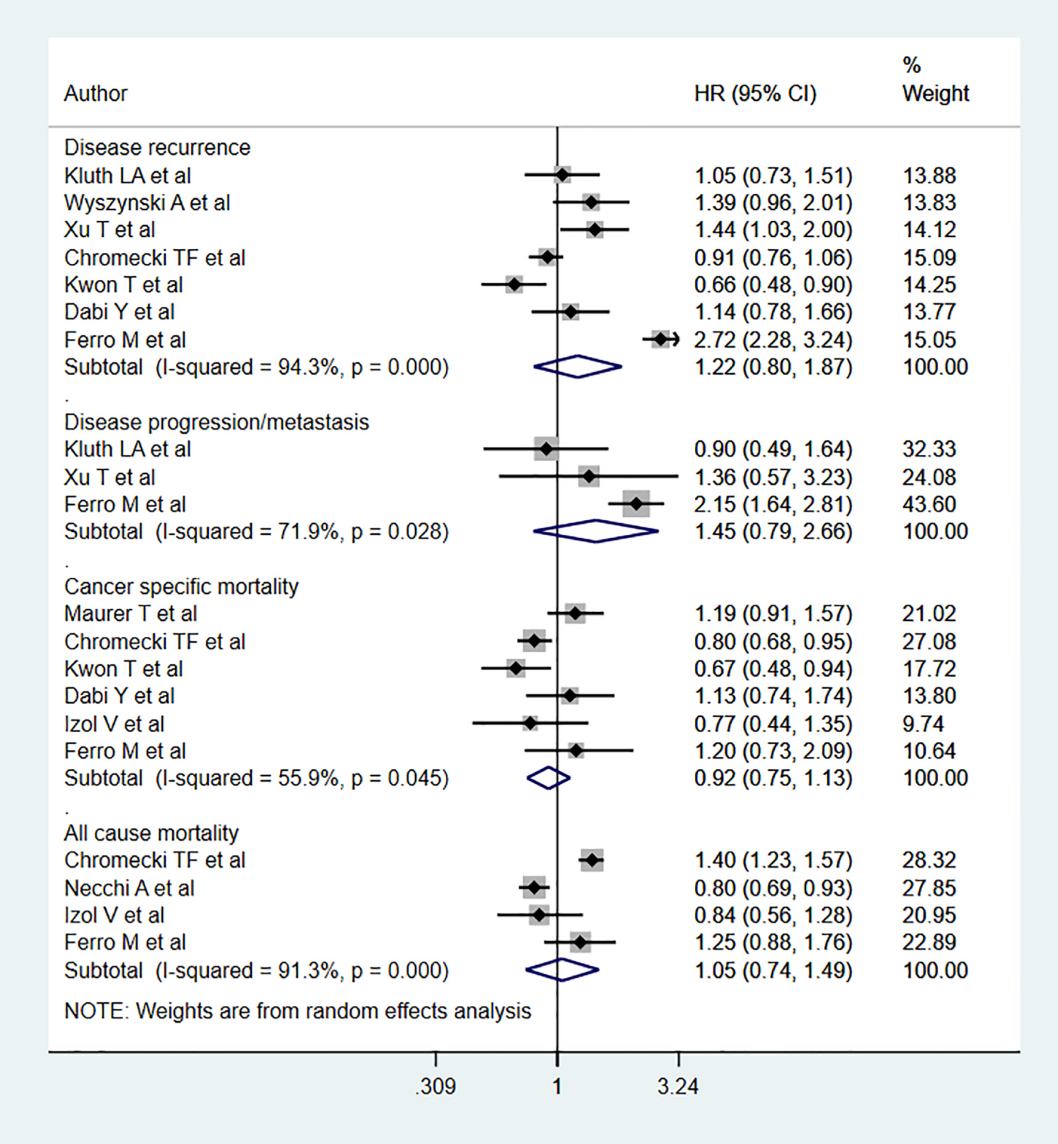
Bladder cancer patient outcomes in overweight and normal BMI patients.

No statistically significant association was noted between BMIs classified as overweight and all-cause mortality (HR 1.05, 95% CI: 0.74, 1.49, n=4) relative to the relationship between normal BMIs and all-cause mortality. Likewise, no association was noted for cancer specific mortality (HR 0.92, 95% CI: 0.75, 1.13, n=6), disease progression (HR 1.45, 95% CI: 0.79, 2.66, n=3), or recurrence (HR 1.22, 95% CI: 0.80, 1.87, n=7) (**Figure 3**). Egger’s test revealed no evidence of publication bias for any of the considered outcomes (P=0.33 for all-cause mortality; P=0.24 for cancer specific mortality, P=0.57 for progression, and P=0.37 for recurrence)

Subgroup analysis showed a decrease risk of cancer-specific mortality for overweight individuals with muscle-invasive bladder cancer (MIBC) (HR 0.77, 95% CI: 0.67, 0.89, n=3) compared to normal BMI counterparts (**Table 2**). All-cause mortality was also decreased for overweight patients with low grade tumors (HR 0.80, 95% CI: 0.69, 0.93, n=1). However, the risk of recurrence increased (HR 1.42, 95% CI: 1.11, 1.81, n=2). No significant differences were noted in any other subgroup analyses (**Table 2**).

**Table 2:** Subgroup analysis for bladder cancer outcomes in overweight patients relative to normal BMI.

	Type of tumor		Grade of tumor		Stage of tumor	
	Non-muscle invasive (NMIBC)	Muscle invasive (MIBC)	Low	High	Early stage (PTa or T1)	Advanced (≥T2)
All-cause mortality	N=2	N=2	N=1	N=3	N=2	N=2
	0.97 (0.63, 1.50)	1.13 (0.69, 1.85)	0.80 (0.69, 0.93)	1.20 (0.91, 1.58)	0.97 (0.63, 1.50)	1.13 (0.69, 1.85)
Cancer specific mortality	N=3	N=3	N=1	N=5	N=2	N=4
	1.18 (0.95, 1.45)	0.77 (0.67, 0.89)	1.19 (0.91, 1.56)	0.84 (0.70, 1.02)	0.86 (0.49, 1.52)	0.96 (0.75, 1.22)
Risk of progression	N=3	—	N=1	N=2	N=3	—
	1.45 (0.79, 2.66)		1.36 (0.57, 3.24)	1.45 (0.62, 3.40)	1.45 (0.79, 2.66)	
Risk of recurrence	N=5	N=2	N=2	N=5	N=5	N=2
	1.48 (0.96, 2.26)	0.80 (0.59, 1.09)	1.42 (1.11, 1.81)	1.15 (0.65, 2.03)	1.31 (0.75, 2.29)	0.95 (0.80, 1.14)

**Figure 4:**
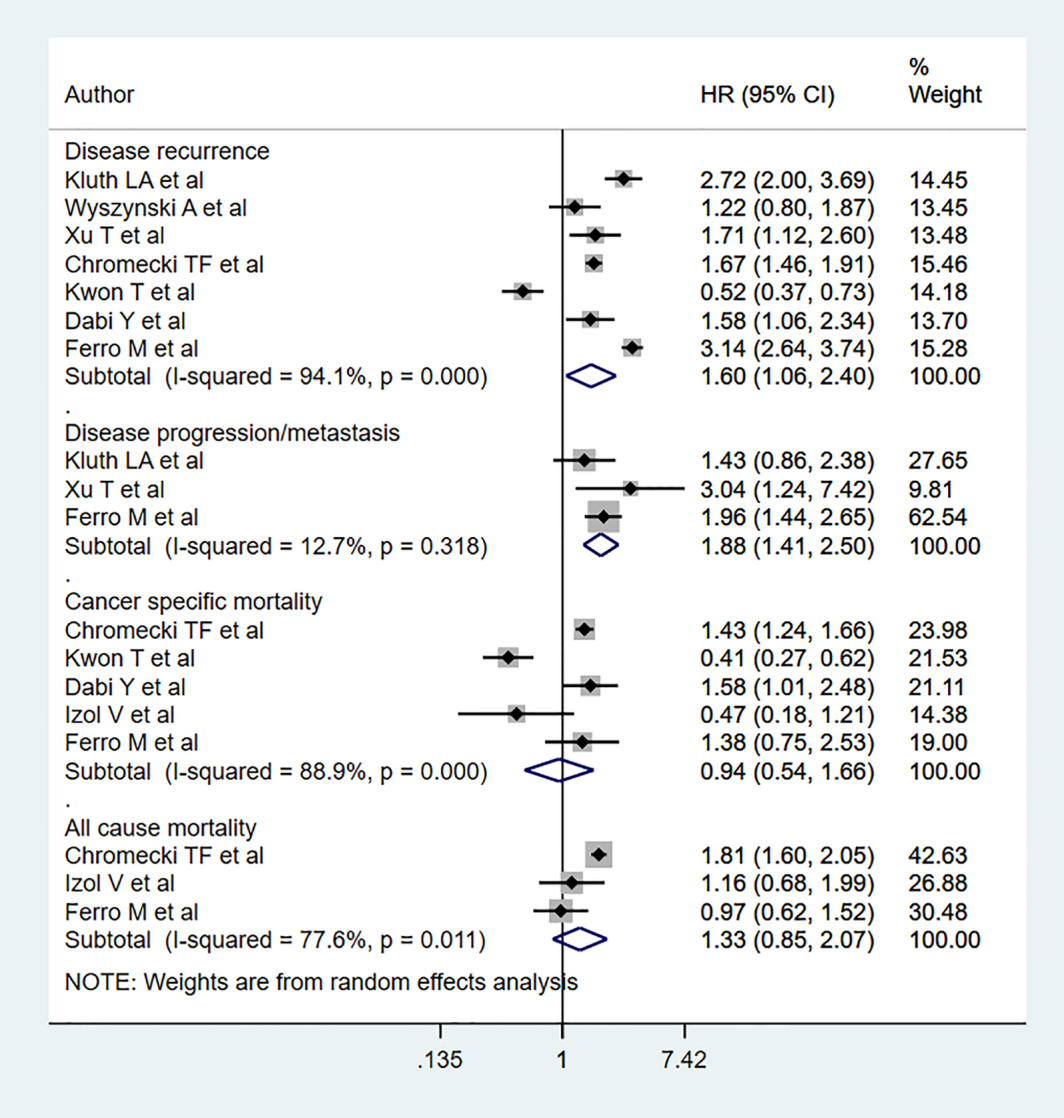
Bladder cancer patient outcomes in obese and normal BMI patients.

Compared to patients with normal BMI, obese patients had elevated risk for disease progression (HR 1.88, 95% CI: 1.41, 2.50, n=3) and recurrence (HR 1.60, 95% CI: 1.06, 2.40, n=7). However, no statistically significant risk change was noted for all-cause mortality (HR 1.33, 95% CI: 0.85, 2.07, n=3) or cancer-specific mortality (HR 0.94, 95% CI: 0.54, 1.66, = 5) (**Figure 4**). Subgroup analysis showed that obese patients with muscle-invasive bladder cancer (MIBC) had elevated risk for all-cause mortality (HR 1.57, 95% CI: 1.04, 2.36, n=2) (**Table 3**). Moreover, obese patients with non-muscle invasive bladder cancer showed elevated risk for cancer specific mortality (HR 1.51, 95% CI: 1.05, 2.16, n=2), disease progression (HR 1.88, 95% CI: 1.41, 2.50, n=3), and recurrence (HR 2.01, 95% CI: 1.39, 2.90, n=5) (**Table 3**). Obese patients also presented elevated risk of progression and recurrence regardless of whether the patient had low- or high-grade cancer. Obese patients with advanced-stage tumors (≥T2) showed higher risk for all-cause mortality (HR 1.57, 95% CI: 1.04, 2.36, n=2) and disease recurrence (HR 1.66, 95% CI: 1.46, 1.89, n=2) (**Table 3**).

**Table 3:** Subgroup analysis for bladder cancer outcomes in obese patients relative to normal BMI.

	Effect size (Hazard ratio; HR) (95% Confidence Interval)					
	Type of tumor		Grade of tumor		Stage of tumor	
	Non-muscle invasive (NMIBC)	Muscle invasive (MIBC)	Low	High	Early stage (PTa or T1)	Advanced (≥T2)
All-cause mortality	N=1	N=2	—	N=3	N=1	N=2
	0.97 (0.62, 1.52)	1.57 (1.04, 2.36)		1.33 (0.85, 2.07)	0.97 (0.62, 1.52)	1.57 (1.04, 2.36)
Cancer specific mortality	N=2	N=3	—	N=5	N=2	N=3
	1.51 (1.05, 2.16)	0.68 (0.25, 1.84)		0.94 (0.54, 1.66)	0.74 (0.22, 2.42)	1.26 (0.83, 1.91)
Risk of progression	N=3	—	N=1	N=2	N=3	—
	1.88 (1.41, 2.50)		3.04 (1.24, 7.44)	1.79 (1.36, 2.37)	1.88 (1.41, 2.50)	
Risk of recurrence	N=5	N=2	N=2	N=5	N=5	N=2
	2.01 (1.39, 2.90)	0.94 (0.30, 2.96)	1.45 (1.04, 2.01)	1.65 (1.01, 2.76)	1.57 (0.80, 3.08)	1.66 (1.46, 1.89)

## Discussion

The current meta-analysis aimed to examine the relationship between bladder cancer outcomes and diabetes or body weight. This study found that diabetic patients had significantly elevated risk for all-cause mortality, cancer specific mortality, disease progression, and recurrence. Obese patients also showed significantly elevated risk for disease progression and recurrence. However, no change in risk for all-cause mortality and cancer specific mortality was noted.

The deleterious influence of diabetes on cancer-related outcomes has been previously documented (41–43). However, the exact underlying mechanisms for how diabetes and elevated BMIs can affect cancer-related outcomes are unclear. Researchers have hypothesized that hyperinsulinemia or hyperglycemia are involved, as chronic hyperinsulinemia or hyperglycemia has been shown to promote tumor cell proliferation and metastasis (44–47). Similarly, elevated levels of insulin-like growth factor (IGF)-1 induces cellular proliferation while inhibiting apoptosis (44–47). Excess adiposity also creates a pro-inflammatory environment, and this may contribute to poorer prognostic outcomes in cancers. Diabetes has also been linked to increased risk for urinary tract infection (UTI), which in turn has been linked to elevated risk for bladder cancer onset, recurrence, and progression (48, 49).

While the findings of the meta-analysis indicate that diabetes is associated with mortality, recurrence and tumor progression, there are many considerations to take into account. First and foremost, we did not explore whether there was a difference between the non-bladder cancer death risk and the bladder cancer death risk in diabetic patients as none of the included studies had non-bladder cancer subjects. Having this analysis would have been important to understand whether presence of diabetes increased the risk of mortality in subjects with bladder cancer, over and above the risk of mortality in the general population or subjects with no bladder cancer. Available evidence indicates that the risk of mortality due to diabetes in the general population is similar to the estimates from the current meta-analysis involving patients with bladder cancer. This may imply that presence of diabetes among subjects with bladder cancer does not significantly increase the risk of mortality, when compared to the general population. However, this finding should not be interpreted as lack of benefit in terms of survival among bladder cancer patients through efforts aimed at better glycemic control. Diabetes is a multifactorial disease where duration, glycosylated hemoglobin levels (HbA1c), glycemic variability, age of patients and sex constitute a cluster with very different impact on clinical peculiarity. In the included studies, majority of the participants had type 2 diabetes and were on oral hypoglycemics. The participants were usually aged more than 60 years of age and majority were males. A growing amount of evidence supports a link between obesity-associated inflammation and cancer incidence and progression (50). Obesity leads to a stage of chronic inflammation with upsurge in inflammatory cytokines leading to an increase the number of cells with tumor-forming capabilities (51). Inflammatory markers such as IL-6, TNF-α, and prostaglandin E2 are all elevated in obese patients. Another important issue is that the levels of leptin are usually higher in patients with obesity. It is well established that leptin induces the expression of pro-inflammatory and pro-tumor cytokines including IL-1, IL-6 and TNF-α (52). The pro-tumor role of leptin is usually due to its role in the promotion of angiogenesis and in enhancing the proliferation and survival of tumor cells. On the other hand, it also inhibits apoptosis, thereby, leading to progression and metastasis (53).

This study highlights the need for close monitoring, supervision and follow up in urinary bladder cancer patients presenting with either elevated BMI and/or diabetes in order to alleviate the risk of mortality, recurrence and disease progression. This study did have several limitations. First, almost all included studies were retrospective, making it difficult to account for any adjustment for potential confounding factors. There is a need for future prospective studies on this issue in order to provide reliable and unbiased evidence. One of the obvious limitations of this meta-analysis is the lack of evidence synthesis on the association of glycemic control (using HbA1c) with the outcomes. This could not be done because of included studies not reporting this association. Further, this was not the primary analysis planned and future research should aim to explore this association. Another limitation relates to the inclusion of multicentric studies and the lack of information concerning protocol harmonization across centres. The study attempted to derive an association of diabetes and BMI with mortality, progression or cancer recurrence. However, it should be noted that a significant overlap between diabetes, obesity, insulin and hypoglycemic agents on cancer outcome could be a major cause of bias in this study. We did not find a statistically significant association between BMI classified as overweight and all-cause mortality. However, there is a limitation to it. There is no unique reference range/operational definition for BMI that was being used to categorize overweight in the included studies. Further, there is a difference in the reference range based on the gender of the participants. While it would have been useful to perform an adjunctive analysis according to sex and related BMI, such an analysis could not be done because of lack of reported gender specific findings in the studies included in the meta-analysis. Finally, retrospective studies largely assessed the presence or absence of diabetes based on medical records/treatment history, and this could result in bias concerning classification. Similarly, different studies used different cut-offs to define “overweight” and “obese”. These discrepancies may lead to inter-study heterogeneity.

## Conclusion

The current meta-analysis suggests that both diabetes and excessive BMI can potentially negatively influence bladder cancer outcomes such as mortality, progression, and recurrence. The risk of mortality due to diabetes in patients with bladder cancer was similar to that in the general population. However, this finding does not undermine the need for better glycemic control in these patients in order to improve survival. Given that retrospective study designs may be subject to certain biases, there is a need for prospective studies investigating this relationship.

## Data Availability Statement 

The raw data supporting the conclusions of this article will be made available by the authors, without undue reservation.

## Author Contributions

YL conceived and designed the study. JT and YL did literature search, analysis and wrote the paper. YL reviewed and edited the manuscript. All authors contributed to the article and approved the submitted version.

## Conflict of Interest

The authors declare that the research was conducted in the absence of any commercial or financial relationships that could be construed as a potential conflict of interest.

## Publisher’s Note

All claims expressed in this article are solely those of the authors and do not necessarily represent those of their affiliated organizations, or those of the publisher, the editors and the reviewers. Any product that may be evaluated in this article, or claim that may be made by its manufacturer, is not guaranteed or endorsed by the publisher.
