# Supplementary material for: Diabetes Mellitus and Obesity as Risk Factors for Bladder Cancer Prognosis: A Systematic Review and Meta-Analysis
Source: Front Endocrinol (Lausanne). 2021 Oct 7;12:699732. doi: 10.3389/fendo.2021.699732 (PMC8529220; doi:10.3389/fendo.2021.699732)
Supplement: Supplementary file 1 [file Table_1.doc]

**Supplementary table 1. Search strategy for relevant studies for inclusion**

| **Search strategy**  #1 (Bladder cancer OR muscle invasive OR non-muscle invasive OR urogenital cancer)  #2 (diabetes OR diabetes mellitus OR glycemic control OR body mass index OR BMI OR overweight OR obese)  #3 (mortality OR death OR recurrence OR progression OR metastasis OR adverse events OR all cause mortality OR cancer specific mortality)  #4 (#1 AND #2 AND #3)  #5 (Addresses[ptyp] OR Autobiography[ptyp] OR Bibliography[ptyp] OR Biography[ptyp] OR pubmed books[filter] OR Case Reports[ptyp] OR Congresses[ptyp] OR Consensus Development Conference[ptyp] OR Directory[ptyp] OR Duplicate Publication[ptyp] OR Editorial[ptyp] OR Systematic reviews OR Meta analysis OR Festschrift[ptyp] OR Guideline[ptyp] OR In Vitro[ptyp] OR Interview[ptyp] OR Lectures [ptyp] OR Legal Cases[ptyp] OR News[ptyp] OR Newspaper Article[ptyp] OR Personal Narratives [ptyp] OR Portraits[ptyp] OR Retracted Publication[ ptyp] OR Twin Study[ptyp] OR Video-Audio Media[ptyp])  #6 (#4 NOT #5) |
| --- |

**Supplementary table 2**. Information on included studies

| **Author (year of publication)** | **Study design** | **Country** | **Participant characteristics** | **Sample size and follow up period** | **Exposure variable of interest** | **Key outcome** |
| --- | --- | --- | --- | --- | --- | --- |
| Rieken M et al (2013) | Retrospective analysis of patient data | Multicentric | Patients had non-muscle invasive bladder cancer with a mean age of 65 (12.7) years and majority were males (>75%). Around 11% of the participants had diabetes and of them, 35.0% were on metformin. Nearly 75% were former or current smokers in both the diabetic and non-diabetic groups  Majority had pathologic stage of PTa (~59%); pathologic grade G3 (~45%) and with no carcinoma in situ (94%); Majority had only one tumour (65%) and tumour size ranged between 1 to 2.9 cm (40%).  All the patients underwent transuretheral resection of bladder (TURB) following which 56% did not received any intravesical therapy and remaining 44% received intravesical therapy with mainly adjuvant BCG | Total sample size of 1117  Median follow up of 64 months | Diabetics compared to non-diabetics | Risk of disease recurrence: HR 1.39 (95% CI: 1.04, 1.86)  Risk of disease progression: HR 2.21 (95% CI: 1.29, 3.77)  Risk of all cause mortality: HR 1.19 (95% CI: 0.81, 1.73) |
| Oh JJ et al (2015) | Retrospective analysis of patient data | South Korea | Patients with non-metastatic urothelial bladder cancer and undergoing open radical cystectomy; majority with no muscle invasion (~90%); majority had no carcinoma in situ (>90%); majority had ≥T2 stage (57%); Majority had pathologic stage of PT2a (~90%); high histological grade (>90%)  Majority were males (>85%); median age was 65.8 years. Only type II diabetics were included; around 60% of the diabetics were on metformin. Data on smoking history of the subjects not provided | Total sample size of 200  Median follow-up duration of 38.3 months | Diabetes status was collected from medical records | Risk of disease recurrence: HR 1.21 (95% CI: 0.78, 1.88)  Risk of disease progression/metastasis: HR 0.85 (95% CI: 0.27, 2.68)  Risk of all-cause mortality: HR 1.28 (95% CI: 1.05, 1.47)  Risk of cancer-specific mortality: HR 1.78 (95% CI: 1.25, 2.29) |
| Faiena I et al (2016) | Retrospective analysis of data from National nationwide inpatient sample | USA | Patients with urothelial bladder cancer and undergoing open radical cystectomy; Majority with muscle invasive bladder ca (?); males (>85%); mean age was 68 years. Data on type of diabetes in the study subjects and their ongoing treatment not provided. Data on smoking history of the subjects not provided | Total sample 90,285 | Diabetic status assigned according to “International Classification of Diseases, Ninth Revision, Clinical Modification” codes | Risk of cancer specific mortality: HR 2.69 (95% CI: 2.02, 3.57)  Risk of post-operative complications: HR 2.07 (95% CI: 1.78, 2.39) |
| Ferro M et al (2020) | Retrospective analysis of patient data | Multicentric | Patients with non-muscle invasive bladder cancer*.* All patients underwent TURB followed by adjuvant intravesical therapy with BCG.  Mean age of 70.3 years; majority were males (>80%); All had primary tumor of T1 G3; Majority had no carcinoma in situ (~85%); Majority had tumour size of ≥3 cm (~65%); more than half of the subjects had one tumour (~55%). All the patients with diabetes were type II diabetics. Nearly 75% were smokers in those with diabetes and 70.8% in non-diabetic group. | Total sample size of 1172  Median follow-up duration of 47 months | Diabetes diagnosed using American Diabetes Association guidelines; both of HbA1c criteria and plasma blood glucose (either fasting or the 2-hour plasma glucose value after a 75-g oral glucose tolerance test) | Risk of disease recurrence: HR 1.41 (95% CI: 1.32, 1.51)  Risk of disease progression/metastasis: HR 1.35 (95% CI: 1.12, 1.64)  Risk of cancer-specific mortality: HR 1.41 (95% CI: 1.20, 1.66) |
| Ahn JH et al (2016) | Retrospective analysis of patient data | South Korea | Patients with non-muscle invasive bladder cancer*.* All patients underwent TURB; intravesical therapy was additionally done in 70%  Median age of 64.6 years; majority were males; Majority had stage Ta (68%) and had low grade carcinoma (62%); Majority had tumour size of <3 cm (~70%); more than half of the subjects had 1-2 tumour (~55%). All the patients with diabetes were type II diabetics and majority were on metformin. Similar proportion of subjects in diabetic and non-diabetic group were smokers (48%) | Total sample size of 645  Median follow-up duration of 46 months | Diabetes diagnosed either through history of diabetes or medical therapy along with pre-operative elevated fasting glucose levels (>126 mg/dl) | Risk of disease recurrence: HR 1.34 (95% CI: 1.07, 1.68)  Risk of disease progression/metastasis: HR 1.77 (95% CI: 1.16, 2.68)  Risk of cancer-specific mortality: HR 1.79 (95% CI: 1.13, 2.84) |
| Wei-Lun H et al (2020) | Retrospective chart review of a prospective database | Taiwan | Patients with non-muscle invasive bladder cancer*.* All patients underwent TURB; intravesical therapy was additionally done in >95%  Median age of 65 years; males (>75%); Tumour stage Ta (60%); carcinoma is situ was absent in majority (~80%); Majority of the tumour were high grade (>60%); majority had 1-2 tumour (>50%) with size <3 cm (~60%). Majority of the patients with diabetes were type II diabetics and majority were on metformin. A total of 37.3% were smokers in diabetic group and 41.3% in non-diabetic group | Total sample size of 287  Median follow-up duration of 45 months | Diabetes diagnosed either through history of diabetes or medical therapy or laboratory based blood glucose levels according American Diabetes Association guidelines | Risk of disease recurrence: HR 1.52 (95% CI: 0.86, 2.69)  Risk of disease progression/metastasis: HR 0.73 (95% CI: 0.20, 2.60)  Risk of cancer-specific mortality: HR 1.11 (95% CI: 0.57, 2.19) |
| Evers J et al (2020) | Retrospective analysis of Nijmegen bladder cancer registry | Netherlands | Patients with non-muscle invasive urothelial bladder cancer*.* All patients underwent TURB with intravesical therapy  Median age of 64 years; males (82%); Tumour stage Ta (70%); carcinoma is situ was absent in majority (~91%); Majority of the tumour were low grade (G1 or G2) (~65%); Majority of the patients had tumours with low risk of progression (~60%); majority had 1 tumour (>50%) with size <3 cm. Majority of the patients (63%) with diabetes were type II diabetics. Nearly 80% of the subjects were smokers (current or former). Data on smoking status not presented for the diabetic and non-diabetic group separately | Total sample size of 1433  Median follow-up duration of 60 months | Diabetes diagnosed either through history of diabetes or reporting of medical therapy for diabetes | Risk of disease recurrence: HR 1.22 (95% CI: 0.98, 1.54)  Risk of disease progression/metastasis: HR 1.16 (95% CI: 0.76, 1.76) |
| Yi-Sheng et al (2014) | Prospective follow up study | Taiwan | Patients with non-muscle invasive urothelial carcinoma (UTUC); Median age of 68 years; Similar proportion of males (48%) and females (52%); Tumour stage >T1 (>50%%); carcinoma is situ was absent in majority (~80%); Majority of the tumour were high grade (~60%); No multifocality in 80%. No data provided on the type of diabetes and the current treatment. Similar proportion of subjects in both diabetic and non-diabetes (20%) | Total sample size of 538  Median follow-up duration of 51 months | Diabetes status was collected from medical records | Risk of disease recurrence: HR 2.10 (95% CI: 1.14, 3.88)  Risk of disease progression/metastasis: HR 1.23 (95% CI: 0.71, 2.16)  Risk of cancer-specific mortality: HR 0.89 (95% CI: 0.50, 1.60)  Risk of all-cause mortality: HR 1.07 (95% CI: 0.68, 1.66) |
| Hwang EC et al (2011) | Retrospective cohort | South Korea | Patients with non-muscle invasive urothelial carcinoma; All the patients underwent transuretheral resection of bladder (TURB) following which 65% received intravesical therapy; Median age of 67 years; Majority were males; Tumour stage >T1 (>50%%); carcinoma is situ was absent in majority; Majority of the tumour were staged Ta (~65%) and were low grade (~63%); Majority had 1-2 tumour (>50%) and tumour size was less than 3 cm (75%). Only type II diabetics were included and were either on oral hypoglycemic agents or insulin. Around 39% were known smokers in the diabetic group and 45% in non-diabetic group | Total sample size of 215  Median follow-up duration of 34 months | Diabetes diagnosed either through history of diabetes or medical therapy along with pre-operative elevated fasting glucose levels (>126 mg/dl) | Risk of disease recurrence: HR 1.60 (95% CI: 1.18, 2.15)  Risk of disease progression/metastasis: HR 7.77 (95% CI: 2.71, 22.3)  Risk of cancer-specific mortality: HR 2.11 (95% CI: 1.40, 3.20) |

**Supplementary table 3**. Definitions and outcomes concerning BMI for included studies

| **Author (year of publication)** | **Study design** | **Country** | **Participant characteristics** | **Sample size and follow up period** | **Exposure variable of interest** | **Key outcome** |
| --- | --- | --- | --- | --- | --- | --- |
| Bi H et al (2019) | Retrospective analysis of data from Bladder cancer database | China | Majority with high grade (80%) *muscle invasive* bladder cancer (66%); median age of 69 years; majority had stage T2-T4. Data on smoking history of the subjects not provided | Total sample size of 152  Follow up period of around 5 years | BMI was categorized into 4 groups including underweight (< 18.5 kg/m2), normal weight (18.5–23.9 kg/m2), overweight (24–27.9 kg/m2), and obese (≥28 kg/m2) based on guidelines for Chinese individuals | *Risk of mortality (ref: underweight)*  Normal weight: HR 0.56 (95% CI: 0.23, 1.36)  Overweight: HR 0.39 (95% CI: 0.14, 1.09)  Obese: HR 0.18 (95% CI: 0.02, 1.63) |
| Kluth LA et al (2013) | Retrospective analysis of data | Multicentric (US,  Germany, France,  Italy, Canada) | Patients had primary *non-muscle invasive* bladder cancer (T1 high grade urothelial carcinoma). Patients treated with transurethral resection of the bladder with or without intravesical  therapy; Median age was 68 years. Data on smoking history of the subjects not provided | Total sample size of 892  Median follow up was 42.8 months | BMI was categorized into 4 groups based on WHO guidelines | *Risk of recurrence*  Continuous: HR 1.07 (95% CI: 1.04, 1.09)  ≥30 vs. <30: HR 2.66 (95% CI: 2.12, 3.32)  25-30 vs. <25: HR 1.05 (95% CI: 0.73, 1.51)  ≥30 vs. <25: HR 2.72 (95% CI: 2.00, 3.69)  *Risk of progression*  Continuous: HR 1.08 (95% CI: 1.04, 1.12)  ≥30 vs. <30: HR 1.49 (95% CI: 1.00, 2.21)  25-30 vs. <25: HR 0.90 (95% CI: 0.49, 1.64)  ≥30 vs. <25: HR 1.43 (95% CI: 0.86, 2.38)  *Risk of all-cause mortality*  Continuous: HR 1.29 (1.20, 1.37)  ≥30 vs. <30: HR 3.15 (95% CI: 1.74, 5.67)  *Risk of cancer specific mortality*  Continuous: HR 1.06 (95% CI: 1.04, 1.09)  ≥30 vs. <30: HR 1.42 (95% CI: 1.06, 1.92) |
| Wyszynski A et al (2014) | Retrospective analysis of data using New Hampshire State Cancer Registry | USA | Patients had primary *non-muscle invasive* bladder cancer with Ta/T1 low grade cancer (74%); Patients treated with transurethral resection of the bladder with or without intravesical  therapy; Median age >50 years; majority were males (75%); had one tumour (72%) and the size of tumour <3 cm (64%); carcinoma in situ absent in >90%. Around 74% of the subjects were current smokers or had a history of smoking | Total sample size of 726  Total follow up period- Median of 6 yrs | Using NIH guidelines, normal BMI was defined as ≤24.9 kg/m^2^; overweight (high BMI) was defined as BMI 25 kg/m^2^ - 29.9 kg/m^2^; obesity was defined as ≥30 kg/m^2^. | *Risk of recurrence*  24.9 to 29.9 vs. ≤24.9: HR 1.39 (95% CI: 0.96, 2.01)  ≥30 vs. ≤24.9: HR 1.22 (95% CI: 0.80, 1.87)  >24.9 vs. ≤24.9: HR 1.33 (95% CI: 0.94, 1.89) |
| Xu T et al (2015) | Retrospective analysis of data | China | Patients with Ta, T1 non-muscle invasive urothelial bladder cancer; mean age of 67 yrs; majority being males (74%); mostly with grade G1 or G2 cancer (85%); majority had single tumour (52%) and size <3 cm (91%); carcinoma in situ was absent >90%. Data on smoking history of the subjects not provided | Total sample size of 403  Total follow up period- Median of 53 months | Patients were divided into three groups: normal weight (BMI < 24 kg/m(2)), overweight (24 kg/m(2) ≤ BMI < 28 kg/m(2)) and obesity (BMI ≥ 28 kg/m(2)). | *Risk of recurrence*  ≥24 to <28 vs. <24: HR 1.44 (95% CI: 1.03, 2.00)  ≥28 vs. <24: HR 1.71 (95% CI: 1.12, 2.60)  *Risk of progression*  ≥24 to <28 vs. <24: HR 1.36 (95% CI: 0.57, 3.23)  ≥28 vs. <24: HR 3.04 (95% CI: 1.24, 7.42) |
| Maurer T et al (2009) | Retrospective analysis of data | Germany | Patients with bladder carcinoma (majority with non-muscle invasive ca.) and underwent radical cystectomy; Majority males (73%); mean age of 68 yrs; pTis–pT2b pN0 cM0 (33%); pT3a–pT4 pN0 cM0 (34.5%). Data on smoking history of the subjects not provided. | Total sample size of 390  Total follow up period- Median of 60 months | BMI calculated preoperatively according to WHO guidelines;  patients were distributed into normal weight (NW, 18.5–24.9 kg/m 2 ), overweight (OW, 25.0–29.9 kg/m 2 ) and obese ( > 30.0 kg/m 2 ) BMI subgroups. | *Risk of mortality (cancer specific)*  Overweight vs. normal weight: HR 1.19 (0.91, 1.57) |
| Chromecki TF et al (2013) | Retrospective analysis of data | Multicentric (Austria, Germany,  Canada, Czech  Republic) | Patients with muscle invasive (68%) urinary bladder cancer and treated with radical cystectomy; majority were males (80%); stage T2-T4 (70%); grade G2-G3 (93%); almost half of the patients had carcinoma in situ (50%). Data on smoking history of the subjects not provided | Total sample size of 4118  Total follow up period- Median of 44 months | The BMI was calculated based on individual weight and height from the clinical data. The BMI was analysed a categorical variable ( <25 vs 25 – 29.9 and ≥ 30 kg/m 2 ) | *Risk of recurrence*  25-29.9 vs. <25: HR 0.91 (95% CI: 0.76, 1.06)  >30 vs. <25: HR 1.67 (95% CI: 1.46, 1.91)  *Risk of cancer specific mortality*  25-29.9 vs. <25: HR 0.80 (95% CI: 0.68, 0.95)  >30 vs. <25: HR 1.43 (95% CI: 1.24, 1.66)  *Risk of all-cause mortality*  25-29.9 vs. <25: HR 1.40 (95% CI: 1.23, 1.57)  >30 vs. <25: HR 1.81 (95% CI: 1.60, 2.05) |
| Bachir BG et al (2014) | Retrospective analysis of data | Canada | Patients with muscle invasive bladder carcinoma and treated with radical cystectomy or radical nephroureterectomy; mean age of around 66 yrs and >75% males; around 90% were high grade (>T2). Around 70% of the subjects were smokers | Total sample size of 847  Total follow up period- Median of 39 months | BMI was estimated at the time of radical surgery and was calculated using the standard definition of body weight (kg)divided by the height squared (m2). Analysis involved using BMI as continuous variable | *Risk of overall mortality:* 1.02 (0.99, 1.04)  *Risk of cancer specific mortality:* 0.99 (0.96, 1.02)  *Risk of recurrence:* 0.98 (0.96, 1.00) |
| Kwon T et al (2014) | Retrospective analysis of data | South Korea | Patients with predominantly muscle invasive bladder carcinoma and treated with radical cystectomy; mean age of around 62 yrs and nearly 90% males; Most had stage ≤T2 (55%) and high grade ca (86%); no carcinoma in situ is majority (76%). Data on smoking history of the subjects not provided. | Total sample size of 714  Total follow up period- Median of 64.1 months | Patients were categorized into three groups according to the BMI cut-off values for Asians recommended by the WHO as follows: normal (BMI <23 kg/m2), overweight (BMI 23–25 kg/m2) and obese (BMI ≥25.0 kg/m2) | *Risk of recurrence*  23-25 vs. <23: HR 0.66 (95% CI: 0.48, 0.90)  >25 vs. <23: HR 0.52 (95% CI: 0.37, 0.73)  *Risk of cancer specific mortality*  23-25 vs. <23: HR 0.67 (95% CI: 0.48, 0.94)  >25 vs. <23: HR 0.41 (95% CI: 0.27, 0.62) |
| Psutka SP et al (2015) | Retrospective analysis of data | USA | Patients with majorly muscle invasive bladder ca. and treated with radical cystectomy and urinary diversion; mean age of 71 yrs; majority males (86%); majority high grade (90%) and stage of ≥T2 (70%). Data on smoking history of the subjects not provided | Total sample size of 515  Total follow up period- Median of 6.3 yrs | BMI calculated according to WHO guidelines; normal weight (18.5–24.9 kg/m2), overweight (25.0–29.9 kg/m 2 ) and obese ( > 30.0 kg/m 2 ) | *Risk of all-cause mortality*  ≥30 vs. <30: HR 0.79 (95% CI: 0.50, 1.26) |
| Dabi Y et al (2016) | Retrospective analysis of data | France | Patients with predominantly non-muscle invasive bladder carcinoma; mean age of around 65 yrs and nearly >75% males; Most had stage ≥T2 (65%) and high grade ca (95%); carcinoma in situ (~50%). Data on smoking history of the subjects not provided | Total sample size of 701  Median follow up period of 45 months | BMI was defined as the weight in kilograms divided by the square of the height in meters (kg/m2); normal (18 ≤ BMI < 25 kg/m2), overweight (25 < BMI < 30 kg/m2), and obese patients (BMI ≥ 30 kg/m2). | *Risk of recurrence*  >25-30 vs. 18-25: HR 1.14 (95% CI: 0.78, 1.66)  >30 vs. 18-25: HR 1.58 (95% CI: 1.06, 2.34)  *Risk of cancer specific mortality*  >25-30 vs. 18-25: HR 1.13 (95% CI: 0.74, 1.74)  >30 vs. 18-25: HR 1.58 (95% CI: 1.01, 2.48) |
| Hafron J et al (2005) | Retrospective analysis of records | USA | Patients with muscle invasive bladder carcinoma undergoing radical or partial cystectomy; mean age of around 65 yrs and nearly 80% males; Most had stage ≥T2 (60%) and high grade. Around 70% of the subjects had a history of smoking | Total sample size of 288  Median follow up period of 39 months | BMI was calculated on the day of surgery. Standard WHO definitions of BMI were used (normal—less than 25 kg/m2, overweight—25 to 29.9 kg/m2 and obesity—30 kg/m2 or greater) | *Risk of all-cause mortality*  ≥30 vs.≤29.9: HR 0.87 (95% CI: 0.71, 1.06) |
| Leiter A et al (2016) | Retrospective analysis of records | Multicentric study (US,  Germany, Greece,  Taiwan) | Patients with muscle invasive bladder cancer; mean age of around 66 yrs; majority were males (around 75%); majority with tumour grade of ≥T2. Data on smoking history of the subjects not provided. | Total sample size of 537  Follow up period of around 5 years | BMI was classified according to WHO criteria (<18.5 underweight, 18.5–24.99 normal weight, 25–29.99 overweight, >30 obese) | *Risk of all-cause mortality*  18.5-24.99 vs. <18.5: HR 0.97 (95% CI: 0.59, 1.59)  25-29.99 vs. <18.5: HR 1.19 (95% CI: 0.73, 1.95)  ≥30 vs. <18.5: HR 1.08 (95% CI: 0.63, 1.87) |
| Xu X (2016) | Retrospective analysis of data from Cancer Genome Atlas (TCGA) project | USA and Canada | Patients with muscle invasive bladder cancer; mean age of 68 yrs; majority were males; majority ≥T2. Data on smoking history of the subjects not provided | Total sample of 360  Follow up period of around 10 yrs | National Heart, Lung, and Blood Institute's BMI categories of underweight (BMI<18.5), normal weight (18.5 ≤ BMI < 25), overweight (25 ≤ BMI < 30), and obesity (BMI ≥ 30). | *Risk of all-cause mortality*  ≥31.2 vs. <31.2: HR 0.46 (95% CI: 0.26, 0.83)  ≥27 vs. 27: HR 0.77 (95% CI: 0.51, 1.17) |
| Necchi A et al (2017) | Retrospective analysis of data | Multicentric (US,  Canada, Europe,  Israel) | Patients with non-muscle invasive urothelial bladder cancer with median age of 67yrs and majority were males (around 80%). Around 65% of the subjects had a smoking history or were current smokers | Total sample of 1020  Median follow up period of 31.6 months | Not provided | *Risk of all-cause mortality*  26.3-29.1 vs. <23.6: HR 0.80 (95% CI: 0.69, 0.93) |
| Izol V et al (2020) | Retrospective data analysis using the bladder cancer database of the Uro-oncology Association | Turkey | Patients with muscle invasive urinary bladder cancer undergoing radical cystectomy; mean age of around 65yrs; majority were males (87%); majority had tumour grade ≥T2 (77%) and high grade (90%); carcinoma in situ was absent is most (87%). >85% of the subjects had a history of smoking | Total sample size of 494  Median follow up period of 24 months | BMI cut-off values recommended by the WHO; Group 1 (normal weight, <25 kg/m2), Group 2 (overweight, 25.0–29.9 kg/m2) and Group 3 (obese, ≥30 kg/m2) | *Risk of all-cause mortality*  25-29.9 vs. <25: HR 0.84 (95% CI: 0.56, 1.28)  >29.9 vs. <25: HR 1.16 (95% CI: 0.68, 1.99)  Risk of cancer specific mortality  25-29.9 vs. <25: HR 0.77 (95% CI: 0.44, 1.35)  >29.9 vs. <25: HR 0.47 (95% CI: 0.18, 1.21) |
| Ferro M et al (2018) | Retrospective analysis of data | Multicentric | Patients with primary T1G3 non-muscle invasive bladder cancer receiving TURB with adjuvant BCG; mean age of 70 yrs; majority males (80%); most had single tumour and size ≥3 cm (63%); no carcinoma in situ (85%). >60% of the subjects had a history of smoking | Total sample size of 1155  Median follow up of 48 months | Patients were assigned according to the International Classification of adults to the underweight, overweight and obesity group according to the BMI; underweight (BMI < 18.5), normal weight (BMI 18.5–24.99), overweight (BMI 25–29.99) and obese (BMI ≥ 30) categories | *Risk of recurrence*  25-29.9 vs. 18.5-24.9: HR 2.72 (95% CI: 2.28, 3.24)  ≥30 vs. 18.5-24.9: HR 3.14 (95% CI: 2.64, 3.74)  *Risk of progression*  25-29.9 vs. 18.5-24.9: HR 2.15 (95% CI: 1.64, 2.81)  ≥30 vs. 18.5-24.9: HR 1.96 (95% CI: 1.44, 2.65)  *Risk of all-cause mortality*  25-29.9 vs. 18.5-24.9: HR 1.25 (95% CI: 0.88, 1.76)  ≥30 vs. 18.5-24.9: HR 0.97 (95% CI: 0.62, 1.52)  *Risk of cancer specific mortality*  25-29.9 vs. 18.5-24.9: HR 1.20 (95% CI: 0.73, 2.09)  ≥30 vs. 18.5-24.9: HR 1.38 (95% CI: 0.75, 2.53) |

**Supplementary table 4. Study bias assessment using the adapted Ottawa-Newcastle Risk of Bias Assessment tool (studies investigating diabetes)**

|  | Rieken M et al | Oh JJ et al | Faiena I et al | Ferro M et al | Ahn JH et al | Wei-Lun H et al | Evers J et al | Yi-Sheng et al | Hwang EC et al |
| --- | --- | --- | --- | --- | --- | --- | --- | --- | --- |
| Representativeness/appropriateness of participant selection  Random or consecutive recruitment=Y  Convenience sample=N  Not reported or unclear | Y | Y | Y | Y | Y | Y | Y | Y | Y |
| Control for baseline differences in cohorts  Similarity of groups at baseline or adjustment in analyses=Y  No attempt to control or adjust=N  Not reported=NR | Y | Y | N | Y | Y | Y | Y | Y | N |
| Loss to follow-up  Explanation provided for loss of participants and/or intention to treat=Y  No explanation =N | Y | Y | N | Y | Y | Y | Y | Y | Y |
| Masking of exposure to outcomes assessor  Description of masking=Y  No masking or no description =N | Y | Y | Y | Y | Y | Y | Y | Y | Y |
| Ascertainment of condition  Description of ascertainment/diagnostic criteria=Y  No description or patient self-report=N | Y | Y | Y | Y | Y | N | Y | Y | Y |
| Documentation of other treatment modalities  Documentation=Y  No documentation=N | Y | Y | Y | N | Y | Y | Y | N | Y |
| Extent to which valid outcomes are described  Adequate description of outcome=Y  Insufficient detail regarding outcome or follow-up time=N | Y | Y | Y | Y | Y | Y | Y | N | Y |
| Prespecification of harms, mode of harms collection  Description of a list of harms assessed or monitoring=Y  No such description or passive harms collection=N  No adverse events reported=NA | Y | Y | Y | N | Y | N | Y | N | Y |
| Financial Conflict of interest (COI)  Funding source reported=Y  Funding source not reported=N | N | Y | Y | N | Y | N | Y | N | Y |

**Supplementary table 2 continued. Study bias assessment using the adapted Ottawa-Newcastle Risk of Bias Assessment tool (studies investigating BMI)**

|  | Bi H et al | Kluth LA et al | Wyszynski A et al | Xu T et al | Maurer T et al | Chromecki TF et al | Bachir BG et al | Kwon T et al | Psutka SP et al | Dabi Y et al | Hafron J et al | Leiter A et al | Xu X et al | Necchi A et al | Izol V et al | Ferro M et al |
| --- | --- | --- | --- | --- | --- | --- | --- | --- | --- | --- | --- | --- | --- | --- | --- | --- |
| Representativeness/appropriateness of participant selection  Random or consecutive recruitment=Y  Convenience sample=N  Not reported or unclear | Y | Y | Y | Y | Y | Y | Y | Y | Y | Y | Y | N | Y | Y | Y | Y |
| Control for baseline differences in cohorts  Similarity of groups at baseline or adjustment in analyses=Y  No attempt to control or adjust=N  Not reported=NR | Y | Y | Y | Y | Y | Y | Y | Y | Y | Y | Y | N | Y | Y | Y | Y |
| Loss to follow-up  Explanation provided for loss of participants and/or intention to treat=Y  No explanation =N | Y | Y | Y | Y | N | Y | Y | Y | Y | Y | Y | Y | Y | Y | N | Y |
| Masking of exposure to outcomes assessor  Description of masking=Y  No masking or no description =N | Y | Y | Y | N | Y | Y | Y | Y | Y | Y | N | Y | Y | Y | Y | Y |
| Ascertainment of condition  Description of ascertainment/diagnostic criteria=Y  No description or patient self-report=N | Y | Y | Y | Y | Y | Y | Y | Y | Y | Y | Y | N | Y | Y | Y | Y |
| Documentation of other treatment modalities  Documentation=Y  No documentation=N | Y | Y | Y | N | Y | Y | Y | N | Y | Y | Y | Y | Y | Y | Y | Y |
| Extent to which valid outcomes are described  Adequate description of outcome=Y  Insufficient detail regarding outcome or follow-up time=N | Y | Y | Y | Y | Y | N | Y | Y | Y | Y | Y | Y | N | Y | Y | Y |
| Prespecification of harms, mode of harms collection  Description of a list of harms assessed or monitoring=Y  No such description or passive harms collection=N  No adverse events reported=NA | Y | Y | Y | N | Y | Y | N | N | Y | Y | Y | Y | Y | Y | Y | Y |
| Financial Conflict of interest (COI)  Funding source reported=Y  Funding source not reported=N | Y | N | Y | Y | Y | Y | N | Y | Y | N | Y | Y | Y | Y | Y | Y |
